# 16 Circulating Cytokine Ratios Demonstrate the Balance of the Pro and Anti-Inflammatory Response After Burn Injury

**DOI:** 10.1093/jbcr/irae036.016

**Published:** 2024-04-17

**Authors:** Tuan D Le, Desiree Pinto, Anthony Pusateri, Melissa M McLawhorn, Lauren T Moffatt, Jeffrey W Shupp

**Affiliations:** MedStar Washington Hospital Center, Grand Prairie, Texas; MedStar Washington Hospital Center, Washington, DC; Naval Medical Research Unit San Antonio, JBSA-Fort Sam Houston, San Antonio, Texas; Firefighters’ Burn and Surgical Research Laboratory, Washington, DC; MedStar Health, Georgetown University School of Medicine, Washington, DC; MedStar Washington Hospital Center, Grand Prairie, Texas; MedStar Washington Hospital Center, Washington, DC; Naval Medical Research Unit San Antonio, JBSA-Fort Sam Houston, San Antonio, Texas; Firefighters’ Burn and Surgical Research Laboratory, Washington, DC; MedStar Health, Georgetown University School of Medicine, Washington, DC; MedStar Washington Hospital Center, Grand Prairie, Texas; MedStar Washington Hospital Center, Washington, DC; Naval Medical Research Unit San Antonio, JBSA-Fort Sam Houston, San Antonio, Texas; Firefighters’ Burn and Surgical Research Laboratory, Washington, DC; MedStar Health, Georgetown University School of Medicine, Washington, DC; MedStar Washington Hospital Center, Grand Prairie, Texas; MedStar Washington Hospital Center, Washington, DC; Naval Medical Research Unit San Antonio, JBSA-Fort Sam Houston, San Antonio, Texas; Firefighters’ Burn and Surgical Research Laboratory, Washington, DC; MedStar Health, Georgetown University School of Medicine, Washington, DC; MedStar Washington Hospital Center, Grand Prairie, Texas; MedStar Washington Hospital Center, Washington, DC; Naval Medical Research Unit San Antonio, JBSA-Fort Sam Houston, San Antonio, Texas; Firefighters’ Burn and Surgical Research Laboratory, Washington, DC; MedStar Health, Georgetown University School of Medicine, Washington, DC; MedStar Washington Hospital Center, Grand Prairie, Texas; MedStar Washington Hospital Center, Washington, DC; Naval Medical Research Unit San Antonio, JBSA-Fort Sam Houston, San Antonio, Texas; Firefighters’ Burn and Surgical Research Laboratory, Washington, DC; MedStar Health, Georgetown University School of Medicine, Washington, DC

## Abstract

**Introduction:**

Burn injury results in a significant inflammatory response. The pro and anti-inflammatory responses in burn patients are typically studied independently with an emphasis on proinflammation, and as a staged process occurring during specific time frames after burn injury. In other disease processes, the interactions of these systems have shown to be more significant. This study connects the inflammatory responses by developing cytokine protein ratios. We predict that ratios will further characterize the inflammatory response after burn injury and that an unbalanced response will be associated with mortality.

**Methods:**

This prospective observational study enrolled patients admitted to a regional burn center. Patient demographics, injury characteristics, and blood samples of cytokine protein levels (IL-6, IL-10, TNF-α), were collected at admission. IL-6 and TNF-α were considered pro-inflammatory and IL-10 was considered anti-inflammatory. Likelihoods of mortality were calculated using logistic regression for cytokine protein levels at admission. The optimal cut-off values for IL-6 and IL-10 were obtained by logistic regression and area under the curve (AUC) analysis with the Youden index. These cut-off points were then used to develop four pro/anti-inflammatory groups: low IL-6/low IL-10, low IL-6/high IL-10, high IL-6/low IL-10, and high IL-6/high IL-10. Rates of mortality were then compared among these groups.

**Results:**

Of 158 patients, 115 met inclusion criteria. Most patients were male (68.7%), with a median age of 40 and a median TBSA of 11.8%. Overall mortality was 11.3%. The AUC of IL-6 (0.893), IL-10 (0.892), and TNF-α (0.812) revealed all cytokines as excellent predictors of mortality individually. When IL-6 and IL-10 levels were combined, the ability to predict mortality was not improved (AUC 0.885). Based on the AUC and Youden index, the optimal cutoff point for IL-6 was 10.5ng/dL (sensitivity 99.9%, specificity 68.6%), and IL-10 was 8.5ng/dL (sensitivity 84.6%, specificity 84.3%). Most of our patients (52%) were in the low IL-6/low IL-10 group and none of these patients died. A high IL-6/ low IL-10 was associated with a 15.8% mortality rate and a high IL-6/ high IL-10 was associated with a 38.5% mortality rate (p < 0.0001). No patient was in the low IL-6/ high IL-10 group.

**Conclusions:**

Both the pro and anti-inflammatory systems are stimulated following burn injury and their balance analyzed through cytokine ratios is associated with mortality. At admission, patients with a balanced, but significantly elevated pro and anti-inflammatory response have the highest mortality rates.

**Applicability of Research to Practice:**

A balanced, but elevated inflammatory response immediately following burn injury demonstrates a pathologic response. Cytokine ratios may be useful as early biomarkers for disease progression after burn injury.

